# Excision of a huge adrenal pheochromocytoma resembling a pancreatic tumor: A case report

**DOI:** 10.1016/j.ijscr.2020.11.146

**Published:** 2020-12-02

**Authors:** Hideki Kogo, Hideaki Takasaki, Yoshinori Sakata, Yoshiharu Nakamura, Hiroshi Yoshida

**Affiliations:** a Department of Surgery, Nippon Medical School Tama-Nagayama Hospital, Tokyo, Japan; b Department of Surgery, Kamisu Saiseikai Hospital, Ibaraki, Japan; c Department of Gastrointestinal and Hepato-Biliary-Pancreatic Surgery, Nippon Medical School, Tokyo, Japan

**Keywords:** Case report, Huge adrenal tumour, Pancreatic tumour, Pheochromocytoma

## Abstract

**Introduction:**

Pheochromocytoma is a rare disease. Adrenal gland tumors make intraoperative blood pressure control difficult. If the tumor is large, it may need to be differentiated from pancreatic tumors and may require combined resection of other organs.

**Presentation of case:**

A 73-year-old Japanese woman presented to our hospital with abdominal pain. Computed tomography revealed a left abdominal tumor of 15 cm diameter. Magnetic resonance imaging, abdominal ultrasonography, and fractionated catecholamine test results led to strong suspicions of pheochromocytoma. A surgery was performed to remove the large tumor while controlling the blood pressure in the perioperative period and preparing for the possibility of combined resection of other organs.

**Discussion:**

Left adrenal tumors can be difficult to differentiate from pancreatic tumors if the lesion is large. Pheochromocytoma is a catecholamine-producing tumor, and surgery to remove the tumor can be risky if the blood pressure is not controlled in the perioperative period.

**Conclusion:**

Careful preparations must be made to resect a giant pheochromocytoma. Adrenal tumors should always be considered as a differential diagnosis for any lesion suspected of being a large pancreatic body tail tumor.

## Highlights

• Adrenal gland tumors make intraoperative blood pressure control difficult.
• Left adrenal tumors can be difficult to differentiate from large pancreatic tumors.
• Adrenal tumors should be considered as a differentiator of large pancreatic tumors.

## 1 Introduction

Pheochromocytoma is a rare disease. It is known to occur in <0.2% of patients with hypertension [1,2]. The annual incidence of pheochromocytoma is estimated at 0.8 per 100,000 people [3]. The present case is one of the rarest cases, with large tumors measuring up to 15 cm in diameter [4,5].

Since pheochromocytoma cases are based on a state of excess catecholamine production, fluctuations in blood pressure can make surgery dangerous if preparations are not made preoperatively to control blood pressure and circulating blood flow [4,[6], [7], [8]]. Therefore, preoperative, intraoperative, and postoperative preparations for blood pressure control are necessary [4,8].

If the left adrenal tumor is large, the preoperative preparation and treatment must be fully explained, as reports have described patients who required a combined resection of the distal pancreas or left kidney for tumor resection [4,7].

Furthermore, if the left adrenal tumor is large, it may be difficult to differentiate from a pancreatic tumor during diagnosis [[7], [8], [9]]. In the present study, we report a rare case of a large pheochromocytoma wherein the patient was carefully prepared for surgery.

## 2 Presentation of case

A 73-year-old Japanese woman presented to our outpatient clinic with a chief complaint of abdominal pain. She had a history of hypertension. During the initial examination, her vital signs were stable. Her consciousness was clear, and physical examination revealed a flat and soft abdomen without tenderness.

**Fig. 1:**
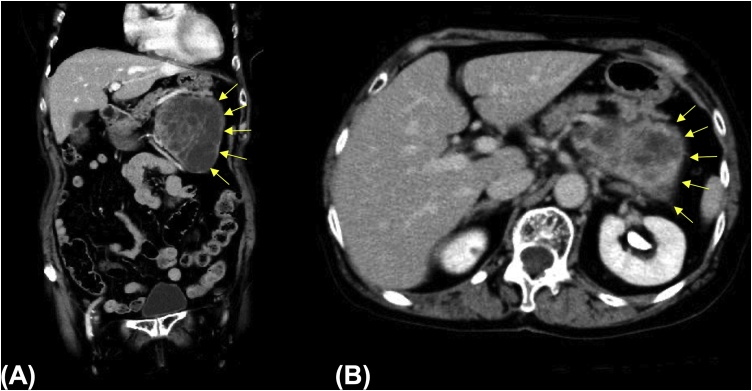
Enhanced computed tomography image of the abdomen. Coronal (**A**) and (**B**) images showing a giant tumor measuring 15 cm along the greatest dimension.

The laboratory data were as follows: white blood cell count, 6730/μL; C-reactive protein, 0.03 mg/dL; hemoglobin, 10.2 mg/dL; carcinoembryonic antigen, 2.2 ng/mL; and CA 19-9, 11.4 U/mL. The computed tomography (CT) scan of the abdomen showed an abdominal tumor of 15 cm diameter (Fig. 1**A**, **B**).

The patient was closely examined with pancreatic tumor in mind, but the adrenal tumors were also considered as a differential disease. The plasma catecholamine fractionation test results showed a high noradrenaline level (828 pg/mL). This led us to strongly suspect a pheochromocytoma.

We decided to perform a surgical excision. Because of the excess catecholamine secretion in patients with pheochromocytoma, we anticipated intraoperative blood pressure fluctuations during the excision. Therefore, preoperative blood pressure control with alpha blockers and increased circulating blood volume were important. From 2 weeks before to immediately before the surgery, the patient was given an alpha blocker. The target systolic blood pressure was set at 120 mm Hg. To keep the intravascular volume increased, the patient was admitted to the hospital 1 week before surgery and continued to receive saline at 1000 mL/day, until the day before surgery.

### 2.1 Operative findings

**Fig. 2:**
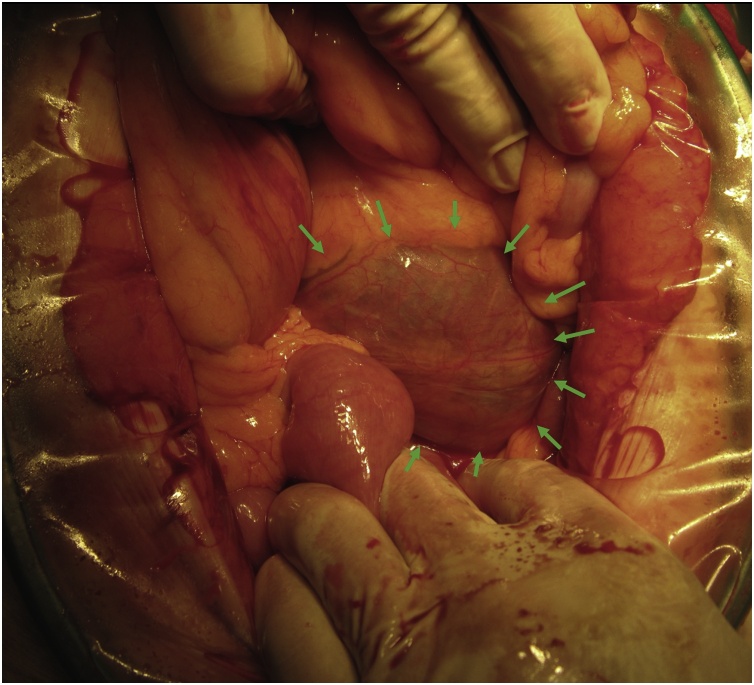
The large retroperitoneal tumor was identified by opening the bursa cavity.

An upper median incision was made. The tumor was identified by opening the bursa cavity (Fig. 2). Owing to the large size of the tumor, the surgical technique was difficult to perform using only a peritoneal approach. Therefore, the Treiz ligament was dissected and approached from the transverse mesenteric side to the retroperitoneum and the tumor was safely removed.

First, the left renal artery/vein was identified and then the left adrenal artery/vein branching from each was identified, ligated, and then dissected. Each of the peripheral vessels around the tumor was ligated and dissected. As a result, the tumor was removed. The area in close proximity to the pancreas was safely dissected.

**Fig. 3:**
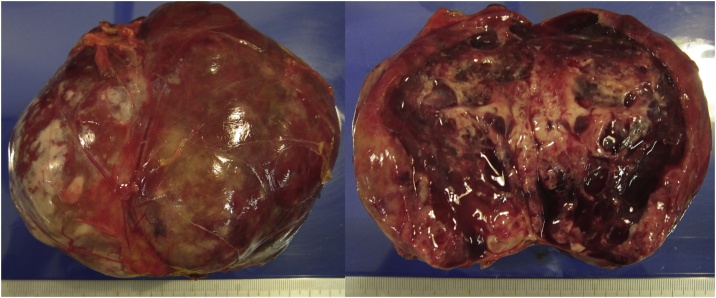
Resected specimen. The histopathological diagnosis was pheochromocytoma of the adrenal gland. No malignancy was confirmed in this sample. The surgical margin was negative.

Intraoperatively, grasping the tumor required careful surgical manipulation, as the systolic blood pressure increased quickly to 200 mm Hg when the tumor was grasped. Blood pressure control required the supervision of a skilled anesthesiologist. The surgical operation time was 144 min, and the blood loss was minimal. The histopathological diagnosis was pheochromocytoma of the adrenal gland (Fig. 3).

### 2.2 Postoperative course

The surgery was uneventful, and the patient progressed postoperatively without difficulty in controlling her blood pressure and was discharged 13 days postoperatively.

## 3 Discussion

Left adrenal tumors can be difficult to differentiate from pancreatic tumors if they are large in size [7,8]. A distal pancreatectomy may also be necessary. Therefore, the operation must be prepared with a pancreatic resection in mind [4,9].

In addition, pheochromocytoma is a catecholamine-producing tumor, and preparations before, during, and after surgery are necessary. Specifically, preoperative saline supplementation to maintain the systemic vascular bed, intraoperative administration of antihypertensive alpha blockers and noradrenaline adjustment, and postoperative noradrenaline tapering are necessary [4,6,8].

This case was also difficult to manage intraoperatively because of the pheochromocytoma and the fluctuations in blood pressure due to intraoperative micro-manipulation [8]. Considering the preoperative and postoperative aspects of pheochromocytoma and the possibility of pancreatic tumor resection, this was a case that required more care than the usual surgery [4,7,8,9].

In addition, adrenal tumors should always be considered as a differentiator of giant pancreatic body/tail tumors. This is because surgery to remove a pheochromocytoma can be risky if blood pressure is not controlled in the perioperative period [[7], [8], [9]]. Adrenal tumors should always be listed as a differential diagnosis for any lesion suspected of being a large pancreatic body tail tumor [7,8].

Pheochromocytoma has three main symptoms, but asymptomatic pheochromocytoma has been occasionally reported and should be suspected even in the absence of symptoms [[4], [5], [6], [7], [8], [9]]. While performing surgery to remove adrenal tumors, the approach is often to remove them from the retroperitoneum [4]. However, in the case of large tumors such as in the present case, the approach is likely through the peritoneal cavity.

To safely and completely resect the large tumor, the pancreas must be identified and the adrenal artery/vein, a feeder vessel, must be ligated and removed. Therefore, we opened the bursa cavity to identify the pancreas and tumor and then dissected the Treiz ligament and approached the transverse mesentery to the retroperitoneum. With the full extent of the massive tumor detailed, we safely removed the tumor. The patient progressed postoperatively without difficulty in controlling her blood pressure and was discharged 13 days postoperatively.

## 4 Conclusion

Careful preparations must be made to resect a giant pheochromocytoma that is difficult to distinguish from a pancreatic tumor. Adrenal tumors should always be considered as a differential diagnosis for any lesion suspected of being a large pancreatic body tail tumor. This case was reported in line with the SCARE guideline [10].

## Declaration of competing interest

The authors declare that they have no competing interests.

## Funding

None.

## Ethical approval

Not applicable.

## Consent

Informed consent was obtained from the patient for the publication of this case report and accompanying images.

## Author contribution

HK: Drafted the manuscript

HK, YS, YN, and HY: performed the operation

HK, YS, HT, YN, and HY: Revised the manuscript

HK, YS, HT, YN, and HY: Read and approved the final manuscript

## Registration of research studies

Not applicable.

## Guarantor

On the behalf of all author I am the guarantor. Hideki Kogo.

## Provenance and peer review

Not commissioned, externally peer-reviewed.

## References

[1] Pacak K., Linehan W.M., Eisenhofer G., Walther M.M., Goldstein D.S. (2001). Recent advances in genetics, diagnosis, localization, and treatment of pheochromocytoma. Ann. Intern. Med..

[2] Stein P.P., Black H.R., Stein P.P., Black H.R. (1991). A simplified diagnostic approach to pheochromocytoma. A review of the literature and report of one institution’s experience. Medicine (Baltimore).

[3] Beard C.M., Sheps S.G., Kurland L.T., Carney J.A., Lie J.T. (1983). Occurrence of pheochromocytoma in Rochester, Minnesota, 1950 through 1979. Mayo Clin. Proc..

[4] Labib M., Ismail A., Elmansy H., Shahrour W., Prowse O., Kotb A. (2019). Adrenalectomy for huge solid pheochromocytoma: a challenging surgery or piece of cake?. J. Surg. Case Rep..

[5] Machairas N., Papaconstantinou D., Papala A., Ioannidis A., Patapis P., Misiakos E.P. (2018). A huge asymptomatic pheochromocytoma. Clin. Case Rep..

[6] Muchuweti D., Muguti E.G., Mbuwayesango B.A., Mungazi S.G., Makunike-Mutasa R. (2018). Diagnostic and surgical challenges of a giant pheochromocytoma in a resource limited setting—a case report. Int. J. Surg. Case Rep..

[7] Antedomenico E., Wascher R.A. (2005). A case of mistaken identity: giant cystic pheochromocytoma. Curr. Surg..

[8] Legocka M.E., Toutounchi S., Pogorzelski R., Krajewska E., Celejewski K., Galazka Z. (2020). Undiagnosed pheochromocytoma presenting as a pancreatic tumor: a case report. Open Med..

[9] Wang H.L., Sun B.Z., Xu Z.J., Lei W.F., Wang X.S. (2015). Undiagnosed giant cystic pheochromocytoma: a case report. Oncol. Lett..

[10] Agha R.A., Borrelli M.R., Farwana R., Koshy K., Fowler A., Orgill D.P., For the SCARE Group (2018). The SCARE 2018 statement: updating consensus surgical case report (SCARE) guidelines. Int. J. Surg..

